# A Cross-Species Analysis of MicroRNAs in the Developing Avian Face

**DOI:** 10.1371/journal.pone.0035111

**Published:** 2012-04-16

**Authors:** Kara E. Powder, Yuan-Chieh Ku, Samantha A. Brugmann, Rose A. Veile, Nicole A. Renaud, Jill A. Helms, Michael Lovett

**Affiliations:** 1 Department of Genetics, Washington University School of Medicine, St Louis, Missouri, United States of America; 2 Division of Plastic Surgery, Division of Developmental Biology, Cincinnati Children's Hospital Medical Center, Cincinnati, Ohio, United States of America; 3 Department of Plastic and Reconstructive Surgery, Stanford University, Stanford, California, United States of America; Ecole Normale Supérieure de Lyon, France

## Abstract

Higher vertebrates use similar genetic tools to derive very different facial features. This diversity is believed to occur through temporal, spatial and species-specific changes in gene expression within cranial neural crest (NC) cells. These contribute to the facial skeleton and contain species-specific information that drives morphological variation. A few signaling molecules and transcription factors are known to play important roles in these processes, but little is known regarding the role of micro-RNAs (miRNAs). We have identified and compared all miRNAs expressed in cranial NC cells from three avian species (chicken, duck, and quail) before and after species-specific facial distinctions occur. We identified 170 differentially expressed miRNAs. These include thirty-five novel chicken orthologs of previously described miRNAs, and six avian-specific miRNAs. Five of these avian-specific miRNAs are conserved over 120 million years of avian evolution, from ratites to galliforms, and their predicted target mRNAs include many components of Wnt signaling. Previous work indicates that mRNA gene expression in NC cells is relatively static during stages when the beak acquires species-specific morphologies. However, miRNA expression is remarkably dynamic within this timeframe, suggesting that the timing of specific developmental transitions is altered in birds with different beak shapes. We evaluated one miRNA:mRNA target pair and found that the cell cycle regulator *p27^KIP1^* is a likely target of miR-222 in frontonasal NC cells, and that the timing of this interaction correlates with the onset of phenotypic variation. Our comparative genomic approach is the first comprehensive analysis of miRNAs in the developing facial primordial, and in species-specific facial development.

## Introduction

Vertebrates exhibit many species-specific morphological differences in craniofacial structures, particularly those derived from the embryonic frontonasal prominence (FNP). In birds these differences are frequently dramatic and result from intense selective pressure to inhabit specific environmental niches. One of the best known examples of this is seen in Darwin's finches [1]. The evolutionary conservation of early vertebrate facial development, coupled with the wide range of different adult beak shapes in birds, has made them an ideal model system for exploring the genetic differences that specify facial variation. In many cases these genetic differences pinpoint genes that are also relevant to human development and craniofacial disorders [2], [3], [4], [5].

Despite differences in the final adult structures, vertebrate embryos look remarkably similar at early stages of facial development [3], [6], [7]. Facial structures then diverge through changes in gene expression and in the delineation of discrete regions of responsiveness in the facial primordia [2], [3], [6], [8], [9], [10]. Vertebrates appear to use essentially the same genetic “tool box” to build facial structures [2], [4], [5], [11], [12], and differences in morphology have been correlated with quantitative, temporal, and/or spatial changes in gene expression [13], [14].

NC cells give rise to all the major tissues and structures of the vertebrate face [15], [16], [17], and in avians have been shown to contain species-specific patterning information [18]. We previously determined that the frontonasal NC cells of the duck, chicken, and quail are morphologically similar at one developmental stage (Hamburger-Hamilton stage 20 [HH20]), but develop different growth trajectories by HH25 [3], [19]. These differences in growth eventually give rise to the broad, flat bill of the duck versus the narrow, deep beak of the chicken and quail. We and others have shown that changes in the Calmodulin, TGF-beta/BMP, and Wnt signaling pathways contribute to these morphological changes in the adult bill shape [2], [3], [8], [9], [10], [20]. By employing genomic methods we previously showed [3] that the expression levels for these pathways and most transcription factors (TFs) are established prior to morphological differentiation. They appear to remain relatively invariant within a given bird species during the critical HH20-HH25 developmental window [3]. Thus, we hypothesized that differential expression of post-transcriptional regulators, such as miRNAs, may also affect morphological alterations of the FNP.

miRNAs have been implicated in a wide range of regulatory roles in development and differentiation, including cellular proliferation, migration, differentiation, apoptosis, and epithelial-mesenchymal transitions (all of which occur in the developing face) [21], [22], [23]. Indeed, conditional knockout of the miRNA processing gene *Dicer* in *Wnt1*-expressing tissues (which include the NC) results in severe craniofacial malformations in mice due to nearly complete ablation of all NC-derived facial bones [22], [24], [25], [26], [27]. NC cells migrate normally in these *Dicer* mutant animals, demonstrating that miRNAs are probably necessary for other processes such as neural crest survival, proliferation, and differentiation during facial development [27]. One previous study [28] described an analysis of some of the miRNAs expressed in one area of the developing vertebrate face. Using microarrays, 70 miRNAs were detected in the developing mouse palate from embryonic stages E12–E14. Many of these miRNAs were developmentally regulated and potentially regulate mRNAs involved in cell proliferation, differentiation, apoptosis, and other processes necessary for normal facial development [28].

In the current study we used deep miRNA sequencing to identify all miRNAs that are expressed in the avian FNP, which gives rise to the upper bill in birds, and to the structures of the upper face in humans. By employing genome-wide bioinformatic approaches we identified 186 miRNAs expressed in frontonasal NC cells of ducks, chickens, and quails. Thirty-five of these are novel orthologs of previously described vertebrate miRNAs and six are newly described avian-specific miRNAs. At least five of this latter group are conserved within all avian species tested from ratites (large flightless birds such as the ostrich) to chickens and songbirds. The majority of the craniofacial miRNAs are differentially expressed between the FNP NC in ducks, quails and chickens. In marked contrast to our previous analyses of TF mRNA gene expression in the FNP [3], we found large changes in miRNA expression between stages when the developing beak is acquiring species-specific morphology.

We also found that the expression of one differentially expressed miRNA, miR-222, was inversely correlated with the protein expression of its known target, p27^KIP1^, during morphological differentiation of the FNP. During this same time period, steady state levels of *p27^KIP1^* mRNA did not change. p27^KIP1^ is a cell cycle inhibitor that remains at lower levels in the duck, but is increased in the chicken FNP. This is consistent with a model in which p27^KIP1^ acts as a modulator of proliferation in NC cells, but in the duck NC is down regulated by miR-222 leading to more sustained cell proliferation.

Our unbiased genome-wide approach is the first analysis of miRNAs in the developing facial primordia, the first comparative investigation of the role of miRNAs in species-specific facial development and the first description of species-specific miRNAs conserved across all avian lineages.

## Results

### Next-Generation sequencing to detect miRNAs in the frontonasal NC cells of chickens, ducks, and quails

**Figure 1:**
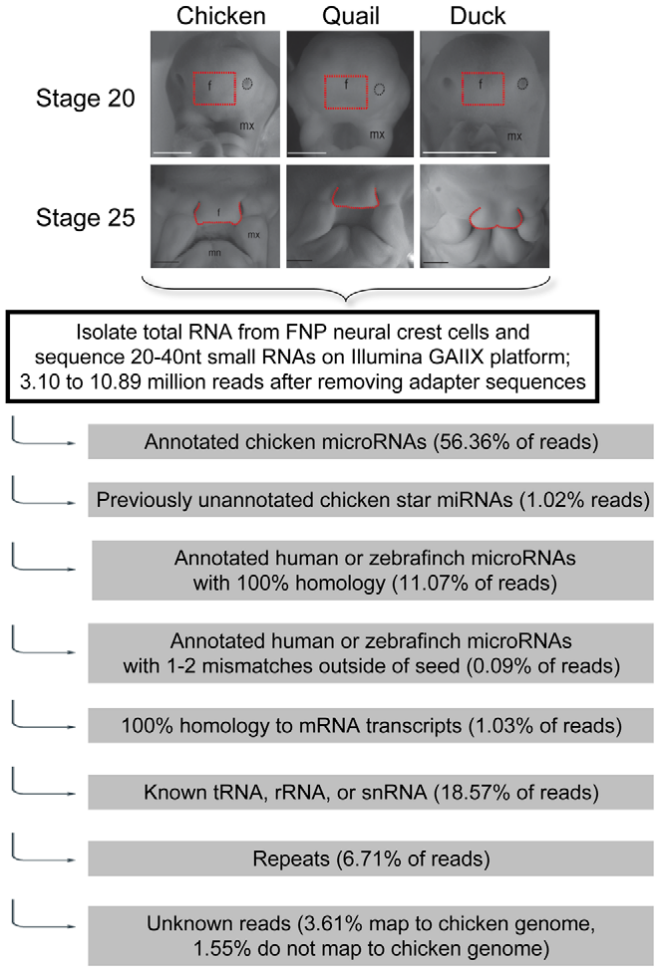
Schematic of analysis pipeline to annotate small RNA reads from frontonasal neural crest cells. At the top are shown representative images of embryonic facial images of the three avian species at either HH stage 20 or 25. The area of dissection is shown in red and is marked with a “f”. The maxillary processes are marked by “mx” and the mandibular prominences by “mn”.

To identify the miRNAs that are expressed in the cranial neural crest we micro-dissected the FNP mesenchyme from 40 duck, quail and chicken embryos at two stages of embryonic development, HH20 and HH25 [3]. Our initial samples were exactly the same RNA preparations employed in our previous study (3). Unlike other facial prominences, FNP mesenchyme consists of a pure population of neural crest cells, rather than a combination of neural crest and mesoderm [29]. HH20 represents a stage at which the facial morphologies of all three species are virtually indistinguishable. By HH25 clear, species-specific morphological differences have arisen [3]. Short RNAs were purified from these cellular populations and analyzed by Next-Generation miRNA sequencing (miRNA-seq) on the Illumina GAIIX platform. Figure 1 illustrates the analysis pathway used to annotate the resulting sequence reads.

Sequencing yielded between 3.10 and 10.89 million reads per sample (after removing adapter reads) with 98.45% of reads mapping to either the chicken genome or to known miRNA orthologs (see below, Figure 1, Figure S1). Technical replicate sequence runs had correlation coefficients of >95% (data not shown). Sequence runs on second biological samples had correlation coefficients of >80%.

The majority of miRNA reads (56.36%) could be clearly identified as representing 122 previously annotated chicken miRNAs (www.mirbase.org, version 16) [30]. However, the computational annotation of chicken miRNAs is clearly incomplete. An additional 1.02% of reads mapped to 31 star (*) strands of known chicken miRNAs for which there were no annotated star activities in current databases (Figure 1 and Figure S1). Star strands are usually found at lower steady state levels than their partner strands, but many have been shown to be biologically active and relevant [31], [32]. These miRNAs are listed with the suffix “ukstar” in Table S1 and Table S2 to indicate that the star strand was previously unknown in the chicken, although in all 31 cases star activity is annotated in other vertebrates. For simplicity, in the text below we refer to all star strands with an asterisk (*) irrespective of whether they are new or previously described.

The *Gallus gallus* genomic sequence (gga3 genome build) is not yet gap-free and may be missing as much as 10% in gapped areas [3], [33], [34]. This raises the possibility that additional miRNAs may not be annotated in miRNA databases [30] or are contained within the sequences that do not map back to the currently available chicken genome. Therefore, we analyzed reads that did not map to known chicken miRNAs to assess whether additional orthologs to known human or zebrafinch miRNAs are present within this set. Another 11.07% of the total reads had 100% sequence identity to 29 human mature miRNAs and 2 zebrafinch miRNAs (Figure 1 and Table S1). These miRNAs are listed in Table S1 and Table S2 with the prefix “hsa” or “tgu” to indicate they are newly described avian orthologs of known human or zebrafinch miRNAs, respectively. We also searched the miRNA sequences for candidate miRNAs that had slight sequence divergence from the known human miRNAs by setting our search algorithms to allow one or two base mismatches outside of the miRNA seed sequence. This identified 4 additional miRNAs that are novel orthologs of human miRNAs (Figure 1). Together these only accounted for 0.09% of total reads. Of the 35 total predicted novel orthologs, only 4 clearly aligned to the available chicken genomic DNA sequence, suggesting that the majority of these miRNAs are not annotated because they fall into gaps in the current chicken genomic assembly. For example, miR-143 and miR-143* have not previously been annotated in the chicken, but we identified multiple reads that matched the human versions of these miRNAs and confirmed expression of miR-143 in avians using qRT-PCR (see below).

In total, 68.54% of sequence reads mapped to chicken, human, or zebrafinch miRNAs (Figure 1 and Figure S1). Within the remaining reads, 1.03% derive from degraded mRNA transcript, 6.71% map to repetitive sequence families, and 18.57% are tRNA, rRNA, or snRNA sequences (Figure 1 and Figure S1). The possibility cannot be discounted that additional data mining of the remaining reads (5.16% of total reads) may yield novel miRNA families.

Overall, by the various analyses and filtering steps described above, we identified 186 mature miRNAs that are detectably expressed in the frontonasal NC cells of the chicken, duck, and quail at a normalized read count of >15 sequences per million mapped reads (PMMR) in at least one sample (Table S1). The 15 PMMR threshold of detection was selected based on the lowest read counts of miRNAs for which we could reproducibly verify trends by qRT-PCR (see below).

### Identification of avian-specific miRNAs

**Figure 2:**
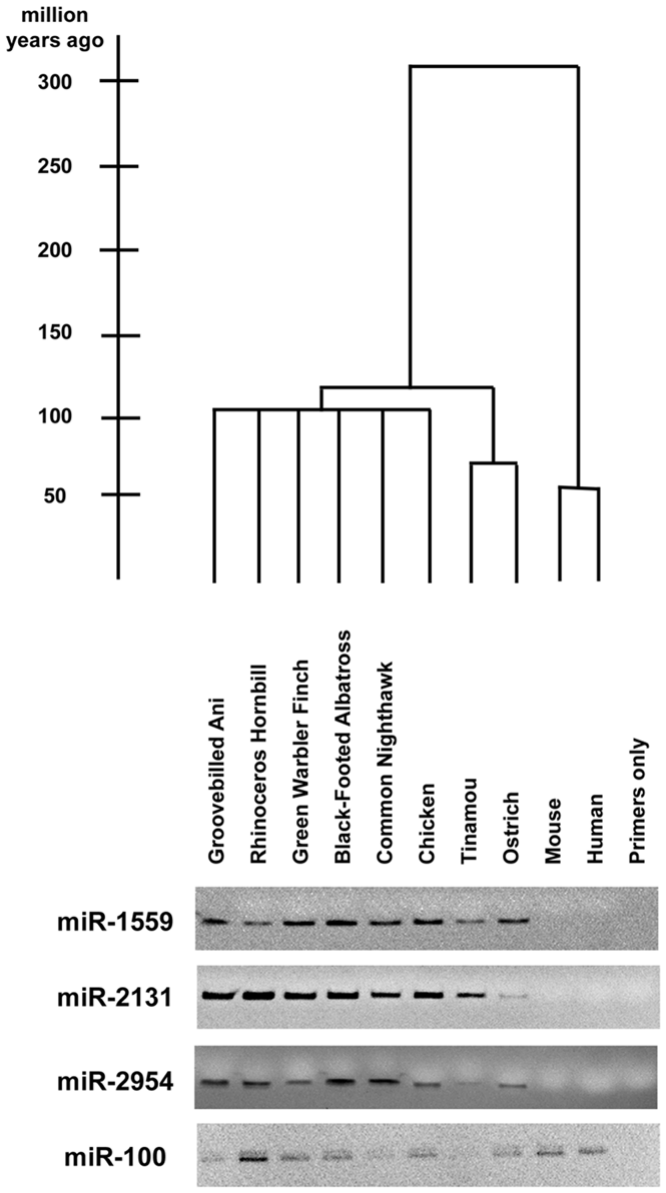
Phylogeny and PCR analysis of avian-specific miRNAs. The top part of this figure shows the phylogenetic tree of the species that we analyzed with the divergence nodes on a scale of millions of years (left). At the bottom are shown the results of gel electrophoresis of PCR products from each genomic DNA for pre-miRNA hairpin precursors for miR-1559, miR-2131, and miR-2954. Indicating that they are conserved across, but are specific to, the avian lineage. The hairpin precursor miR-100 is a positive control that is conserved across all vertebrates examined. The primers only lane is a negative control that lacks genomic DNA.

The studies above represent the first large-scale evaluation of miRNAs in multiple avian species. Therefore, we assessed whether any of the miRNAs that are detectably expressed in the frontonasal NC of chickens, ducks, and quails might be specific to the avian lineage. Birds and mammals shared a last common ancestor ∼310 million years ago [35], and the earliest divergences within birds occurred nearly 120 million years ago (Figure 2) [36].

We compiled a list of six mature miRNAs, mapping to 5 miRNA hairpins, that are only annotated in chicken and zebrafinch in miRBase (www.mirbase.org, version 16) [30], or were identified in other miRNA deep sequencing projects [37], [38]. These sequences are also detectable by sequence alignment searches only in chicken and/or zebrafinch and, as determined above, are expressed in the frontonasal neural crest of the chicken, duck, and quail (Table 1) at relatively high levels. We used PCR to confirm the lineage-specificity of these miRNAs, and found that the hairpin precursors of five of these miRNAs are conserved across, but specific to, the entire avian lineage (∼118.6 million years since last common ancestor) [36], from ratites to galliforms and passerines (Figure 2). These are the first described examples of validated avian-specific miRNAs and join several other examples of miRNAs that have independently evolved within defined species lineages [39], [40], [41], [42], [43].

**Table 1:** Mature miRNAs that are specific to the avian lineage.

mature miRNA	miRBase Accession	miRNA sequence
gga-miR-1451	MIMAT0007324	UCGCACAGGAGCAAGUUACCGC
gga-miR-1559	MIMAT0007416	UUCGAUGCUUGUAUGCUACUCC
gga-miR-2131	MIMAT0011207	AUGCAGAAGUGCACGGAAACAGC
gga-miR-2131*	N/A	CUGUUACUGUUCUUCUGAUG
gga-miR-2954	MIMAT0014448	CAUCCCCAUUCCACUCCUAGCA
gga-miR-2954*	MIMAT0014623	GCUGAGAGGGCUUGGGGAGAGGA

The name, accession number (where available) and mature miR sequence are shown.

As yet, there are no known functions for the five miRNAs that are restricted to the avian lineage (Table 1). These miRNAs may just be an evolutionary novelty, but they may also influence lineage-specific differences. To evaluate potential functionality of these six putative avian-specific microRNAs, we identified potential targets using TargetScan (http://www.targetscan.org/, version 5.1). Many of these predicted targets encode members of developmental pathways (e.g. Fgf, Tgfb, and Wnt signaling), regulate body patterning (e.g. *HOX* genes), or influence chromatin modifications (e.g. *HDAC4*) (Table 2, Table S7). Each predicted mRNA target was further analyzed by ToppGene software (http://toppgene.cchmc.org/) to identify enriched GO annotations. A list of significantly enriched GO annotations for individual avian specific miRNAs is shown in Table S4. These possible miRNA:mRNA target relationships are attractive follow-up candidates for investigating lineage-specific control of these important developmental regulators.

**Table 2:** Selected predicted targets of miRNAs that are limited to the avian lineage.

miRNA	Total targets predicted	Predicted target	Gene description
gga-miR-1451	8	*HOXA10*	homeobox A10
		*ONECUT2*	one cut homeobox 2
gga-miR-1559	2	*HDAC4*	histone deacetylase 4
gga-miR-2131	142	*ACVR2A*	activin A receptor, type IIA
		*ACVR2B*	activin A receptor, type IIB
		*CALM2*	calmodulin 2
		*EN2*	engrailed homeobox 2
		*FGF9*	fibroblast growth factor 9
		*FZD10*	frizzled homolog 10
		*HMGA2*	high mobility group AT-hook 2
		*ONECUT2*	one cut homeobox 2
		*SMAD2*	SMAD family member 2
		*TWIST1*	twist homolog 1
		*ZEB1*	zinc finger E-box binding homeobox 2
		*ZEB2*	zinc finger E-box binding homeobox 2
gga-miR-2131*	44	*CALM2*	calmodulin 2
		*LRP6*	low density lipoprotein receptor-related protein 6
gga-miR-2954	20	*HMGB1*	high-mobility group box 1
gga-miR-2954*	54	*CTNNB1*	beta-catenin
		*LRP6*	low density lipoprotein receptor-related protein 6
		*NUP153*	nucleoporin 153 kDa
		*ONECUT2*	one cut homeobox 2

Targets were predicted using TargetScan (http://www.targetscan.org/) and the seed sequence (nt 2–8) for each of the avian-specific miRNAs. For a complete list of predicted targets see Table S3.

### Dramatic changes in miRNAs occur between developmental stages

In our previous study of these same frontonasal NC samples we measured changes in steady state mRNA levels for ∼2,400 genes involved in developmental signaling pathways and nearly all known and predicted transcription factor genes. Although we found many interesting gene expression differences between species, gene expression was essentially unchanged between HH20 and HH25 within a given species, suggesting that the gene expression profile is established prior to morphological variation [3]. In remarkable contrast to the relatively unchanged pattern of mRNA expression, miRNA expression is dramatically different between the two developmental stages. Of the 186 miRNAs that were detectably expressed, 170 (91%) were differentially expressed by at least 2-fold either between the three species or between the two developmental stages, with fold changes as large as 74-fold (Table S2). The vast majority (132 or 78%) of the 170 miRNAs that were differentially expressed showed changes between the developmental stages in one or more of the species. The specific miRNAs, patterns and trends of miRNA expression are shown in detail in Table S2 and Table S7 and the sections below summarize these trends and relate specific miRNAs to their potential (and in one case, tested) cellular functions.

### miRNAs that regulate stemness, cellular differentiation and epithelia-mesenchyme transitions are differentially regulated between the two developmental stages in all three species

Twelve miRNAs are down-regulated and seventeen are up-regulated from HH20 to HH25 in all three bird species (Table S7). The extent of these changes varies depending upon the particular species. For example, miR-96 is down-regulated at HH25 by 1.81-fold in duck, by 1.84-fold in quail and by 7.35-fold in chicken NC cells. Knockdown of this particular miRNA in zebrafish has previously been shown to result in abnormal cranial cartilage [44]. MiR-302b, miR-302b*, and miR-302c, which are the only members of the 9-member miR-302 family that are detectable at either stage, are down-regulated between 2.3- and 7.8-fold at HH25 in all three species (Table S7). This miRNA family has been previously associated with “stemness.” They are highly expressed in embryonic stem cells, and when induced can reprogram somatic cells into a pluripotent state [45], [46].

Of the seventeen miRNAs that are expressed at higher levels at the later stage of development (HH25) in the chicken, duck, and quail (Table S7) four belong to the miR-30 family (miR-30a-3p, miR-30a-5p, miR-30d*, and miR-30e*). These are up-regulated by between 1.4- to 7.7-fold (Table S7). This family of miRNAs has been previously implicated in promoting mesenchymal-to-epithelial transitions (MET) [47], [48]. While epithelial-to-mesenchymal transitions (EMT) are crucial for neural crest migration [49] and later events of facial development such as lip fusion [50], it is unclear if MET or EMT is occurring in the HH20 to HH25 developmental window. Interestingly, EMT has also been associated with stemness, while MET is associated with cellular differentiation [51], [52], [53]. Thus, up-regulation of the miR-30 family might reflect an increase in cellular differentiation at HH25. In agreement with this, let-7a, let-7a*, let-7c*, let-7d, let-7f, let-7g, let-7i, and let-7k are up-regulated by 1.4- to 27.9-fold at HH25 in all three species, while let-7c is up-regulated at HH25 only in chicken and quail (Table S7). These miRNAs belong to the 19 member let-7 family of miRNAs, the expression of which has been associated with cellular differentiation [54]. In all, 9 of 10 detectable members of the let-7 family are up-regulated in chicken and quail NC by HH25 (Table S7).

Along with let-7c, six additional miRNAs are up-regulated at HH25 only in chicken and quail, but not in duck NC cells. These include miR-30c-2*, miR-129-5p which targets the stem cell regulator *SOX4* [55], [56], the differentiation-promoting miR-137 [57], and the let-7-related miR-100* and miR-125b-2* [58].

A final set of seven miRNAs are only up-regulated in the duck NC compared to chicken and quail after morphological variations are evident at HH25 (Table S7). For example, miR-222 is expressed at similar levels in the duck, chicken, and quail at HH20. However, by HH25, it is down-regulated 1.8-fold in the beaked birds, but remains more highly expressed in duck (Table S7). This miRNA has been shown to down-regulate the cell cycle regulator *p27^KIP1^* in a number of systems, including chicken cell lines [59], [60] (see below for more on this).

### miRNAs that regulate bone formation and Wnt signaling are differentially regulated in the duck compared to the chicken and quail

Twenty-one miRNAs are differentially regulated in the duck compared to chicken and quail at both developmental stages. Six miRNAs with unrelated or unknown functions are expressed at lower levels in NC cells from the flat-billed duck compared to the conical-beaked chicken and quail (Table S7). Fifteen miRNAs are more highly expressed in duck NC cells at both stages (Table S7), including the miR-23a-27a-24-2 cluster, which is negatively regulated by the osteoblast transcription factor RUNX2 [61]. Expression of each of these miRNAs suppresses bone formation and directly down-regulates *SATB2* [61], which has been previously implicated in facial development and associated with morphological variation in the avian beak [3], [62].

Additionally, miR-200a, miR-200b, miR-203, miR-27a, and miR-27b, all of which interact with Wnt signaling components [63], [64], [65], are expressed at 1.5- to 58.9-fold higher levels in duck verses the other species (Table S7). Among this group, miR-200a and miR-200b are remarkable in both showing greater than 50-fold changes in expression between duck and chicken at HH25. We have previously shown that the Wnt pathway regulates regional growth in facial structures and its activation correlates with differences in beak morphology [3]. MiR-200a and 200b have also been shown to regulate MET via direct repression of *ZEB1* and *ZEB2* [66], [67], though, as stated above, it is at present unclear if MET is occurring in the HH20 to HH25 developmental window.

### In situ hybridization and qRT-PCR validate the sequencing data

We confirmed miRNA trends from the sequencing data both *in vitro* and *in vivo*. First, we conducted quantitative real-time polymerase chain reaction (qRT-PCR) on mature miRNAs using a second biological sample of NC cells from HH20 and HH25 ducks and chickens. For nine of ten miRNAs examined, qRT-PCR confirmed expression trends identified by Next-Generation sequencing (Table S1 and Table S5). One miRNA, gga-miR-215, showed a slight discrepancy between qRT-PCR and miRNA-seq data. By sequencing, this miRNA is expressed at higher levels in chicken than duck NC cells at both developmental stages (Table S1). However, by qRT-PCR we only confirmed differential expression at HH20 (Table S5). This miRNA has lower read numbers than most of the other miRNAs confirmed by qRT-PCR, which may account for this discrepancy. Furthermore, absolute changes in miRNA expression did not always agree between sequence data and qRT-PCR as the only commercially available primers for miRNA qRT-PCR are designed from human, not chicken, orthologs. Some sequence differences exist between the miRNAs across that evolutionary period—approximately 310 million years [35]—and this may account for the differences observed between the sequencing and RT-PCR data.

**Figure 3:**
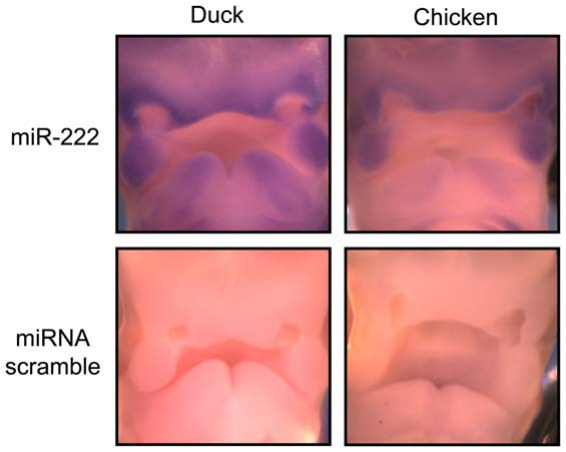
*in situs* validate sequencing data for gga-miR-222 in HH25 chickens and ducks. RNA in situ hybdridizations are shown to HH Stage 25 embryos (cf Figure 1). Upper facial images are shown for duck and chicken comapred to a scrambled control. The lower part of each figure shows the developing mandibular processes. Only the FNP area in the center of each image was the target of dissections (cf Figure 1).

For one of the differentially expressed miRNAs, miR-222, we performed RNA *in situ* to assess the approximate expression level and pattern of the mature miRNA in FNPs from duck and chicken (Figure 3). In both duck and chicken, this miRNA is expressed throughout the facial prominences, but most robustly in the maxillary prominences and around the nasal pits. Though they have similar spatial patterns, miR-222 is expressed at higher levels in the duck, especially within the mandibular prominence, in agreement with our sequencing data (Figure 3).

### Expression of miR-222 correlates with changes in the cell cycle regulator p27^KIP1^ but not with its steady state mRNA levels

Previous studies in multiple species, including chicken, have identified the cell cycle regulator *p27^KIP1^* as one target of miR-222 [59], [60]. miR-222 is expressed at similar levels in the chicken, duck, and quail at HH20. However, by HH25, miR-222 has been down-regulated 1.8-fold in both chicken and quail, but it remains at high levels in duck neural crest cells (Table S7). We sought to determine whether miR-222 may be altering p27 levels in the developing face by measuring levels of p27 protein across a time course using western blots in chicken and duck FNPs from HH17, when NC cell have completed migration into the facial prominences, to HH31, when the adult beak is taking shape [19], [68].

**Figure 4:**
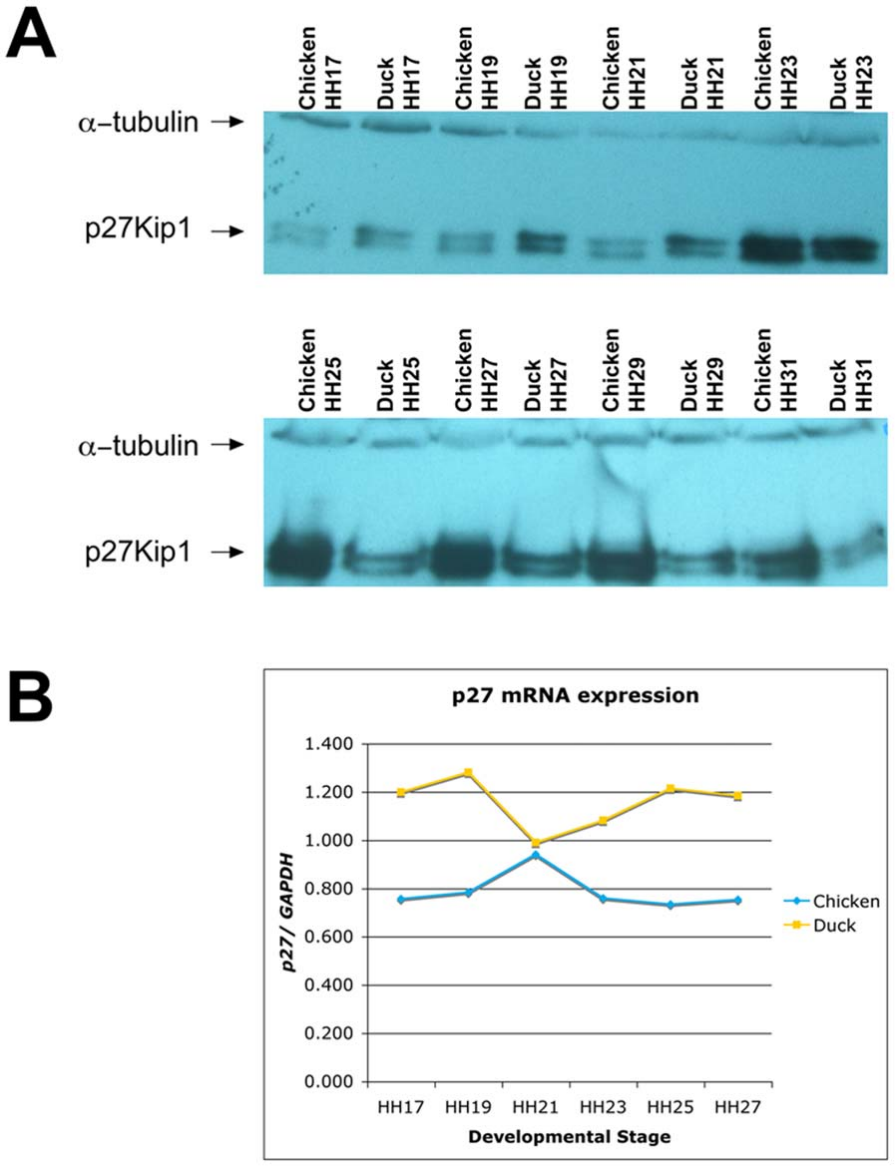
p27^KIP1^ protein, but not mRNA, is differential between birds at the onset of morphological divergence. (A) Western blot analysis of p27 protein (lower doublet) relative to alpha-tubulin loading control (upper band) in a time course of microdissected samples from HH17-HH31 chicken and duck frontonasal prominences. (B) Levels of *p27^KIP1^* mRNA transcripts relative to *GAPDH* control in chicken and duck frontonasal prominences, as measured by qRT-PCR.

From HH17 to HH23, when the duck and chicken embryos are morphologically similar [3], p27 protein is present at similar levels (Figure 4A). However, once the chicken and duck morphologically diverge at HH25, we observed changes in the levels of p27 protein. At HH25, the levels of p27 increase in the chicken but remain relatively constant in duck FNP (Figure 4A). This correlates with the observed increase in miR-222 in the duck (above and Figure 3). The levels of p27 remain at higher levels in chicken FNP through to the end of the time course at HH31.

The increased levels of p27 protein we observed are not accounted for by a corresponding increase in *p27^KIP1^* mRNA levels. By RT-PCR, steady state levels of *p27^KIP1^* transcripts remain relatively constant from HH17 to HH27 in both chicken and duck FNPs (Figure 4B), indicating that post-transcriptional regulation probably accounts for the observed decrease in p27 protein (Figure 4A) and adding another piece of evidence that changes in miR-222 may account for changes in p27 protein.

## Discussion

MiRNAs have an interesting evolutionary history. While the transcription factor and signaling pathway spectrums are largely conserved from sponges to humans [69], [70], miRNAs have been continuously added during the metazoan lineage [71], [72], [73], [74], [75], [76]. The rate of acquisition of new miRNAs has increased at key periods in evolution including the advent of bilaterians, vertebrates, eutherians, and primates [72], [73], [76]. This has lead to the hypothesis that miRNA innovation might have contributed to increases in the morphological complexity of metazoans [72], [75], [76], [77]. Given that this study is the first investigation of miRNAs in multiple avian species, we began by asking whether any of the 186 miRNAs that we detected in the frontonasal neural crest of the chicken, duck, and quail might be specific to the avian lineage. We identified six mature miRNAs that appear to be specific to the avian lineage which has been evolving for nearly 120 million years (Table 1) [36]. We used PCR to confirm that five of these (miR-1559, miR-2131, miR-2131*, miR-2954, and miR-2954*) are conserved across, but are specific to, the entire avian lineage. These are the first described examples of validated avian-specific miRNAs and join several other examples of miRNAs that have independently evolved within defined vertebrate lineages [39], [40], [41], [42], [43]. However, for most species-specific miRNAs it still remains to be determined whether they are an evolutionary dead-ends or have functional roles in development.

Intriguingly, miRNAs might also have a role in species-specific diversification. While humans [78] and mice [79] show negative selection against mutations that destroy conserved miRNA binding sites, the morphologically divergent cichlids of Lake Malawi have increased levels of polymorphism in predicted miRNA binding sites within 3′ UTRs [80]. However, the divergence times within these lineages varies drastically—approximately 370,000 years for humans [81], 23 million years for mice [82], and 1–2 million years for cichlids [83].

**Figure 5:**
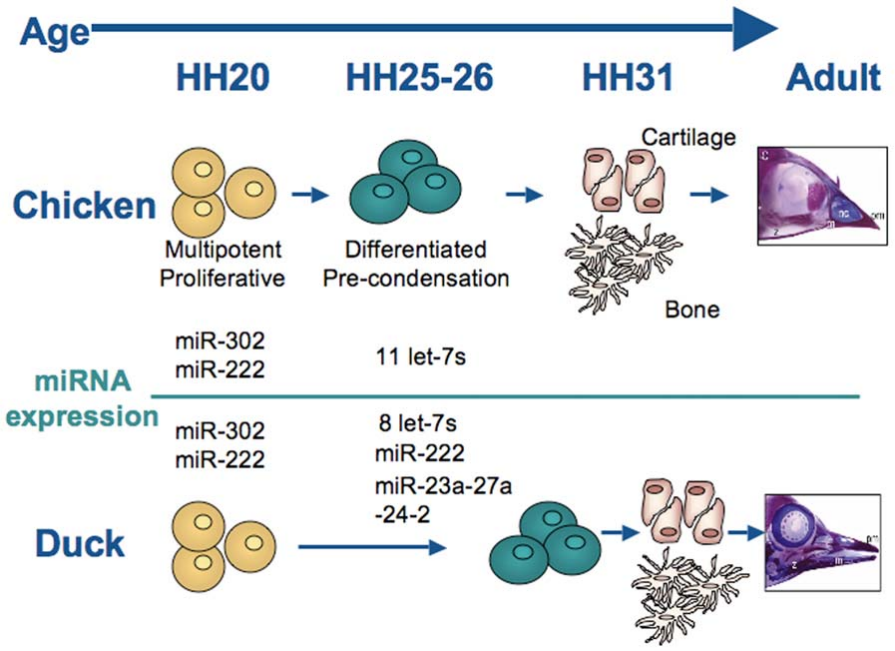
A model of differences in neural crest differentiation and bone formation in duck and chicken. Based on miRNA expression changes, HH20 to HH25 may be the developmental window when multipotent, proliferative neural crest cells (yellow) gain the molecular signatures of differentiation (green) before becoming the cartilage and bones of the face.

In remarkable contrast to the relatively unchanged pattern of mRNA expression we previously observed in these neural crest samples [3], miRNA expression is dramatically different between developmental stages before (HH20) and after (HH25) morphological variation in the beak is evident. The patterns of differentially expressed miRNAs (Table S7) are consistent with the following model (summarized in Figure 5). At HH20, both the chicken and the duck have a multipotent, proliferative NC population that expresses high levels of the miR-302 family as well as high levels of miR-222 (Table S7). These miRNAs promote an undifferentiated fate, in the case of miR-302 [45], [84], and proliferation via repression of *p27^KIP1^*, in the case of miR-222 [59], [60]. By HH25, chicken NC cells have adopted molecular signatures of differentiation. At the same time as the miR-302 family and miR-222 are down-regulated, eleven miRNAs related to the let-7 family are up-regulated, as well as 2 additional miRNAs associated with cellular differentiation (Table S7) [54]. By HH26, chicken facial primordia express molecular markers of the bones and skeleton that will eventually form the adult face [85].

Duck NC cells at HH25 have down-regulated the miR-302 family and up-regulated some of the miRNAs associated with cellular differentiation (i.e. the let-7 family), though not as many as chicken NC (Table S7). However, in contrast to the chicken, duck NC still express high levels of miR-222, and this may act to maintain a higher proliferation rate via continued repression of *p27^KIP1^* [59], [60]. The duck also has higher levels of the miR-23a-27a-24-2 cluster (Table S7). Each of these miRNAs can independently repress the bone-promoting transcription factor *SATB2* [61], [86], and thus the duck may also have a delay in bone formation, as NC cells continue to proliferate.

Taken together, these miRNA changes, including differential expression of let-7, miR-302, and miR-30 families (Table S7), indicate that the HH20 to HH25 developmental window may be a critical transition phase in which multipotent NC cells begin to differentiate to form the various tissues of the face. In addition, given that a number of miRNAs related to let-7 and cellular differentiation are only up-regulated in the chicken and quail at HH25 (Table S7), the timing of this transition may be slightly delayed in the morphologically different duck, perhaps allowing a more prolonged period of proliferation. This is consistent with current theories that differential regions and levels of proliferation can influence the depth, width, and curvature of the beak [9], [10] and that miRNAs function during the transitions between different cellular states [87].

We evaluated one miRNA:mRNA target pair. We speculated that differences in miR-222 levels in the duck versus chicken at HH25 could regulate morphological differences in the beak via its target, the cell cycle regulator p27^KIP1^ [59], [60]. Our hypothesis was that higher levels of miR-222 in HH25 duck, and the resulting decrease of p27 protein, would result in an increased proliferation level. On the other hand, lower miR-222 levels in the beaked chicken and quail could lead to a release of p27 repression and a consequent decrease in proliferation. This model is in agreement with previous analyses that identified higher proliferation levels in HH26-HH31 duck bills compared to chicken beaks [9], [10]. Our analyses of p27 protein and mRNA levels agree with this model: p27 protein is expressed at similar levels in the FNP of the chicken and duck while they are morphologically similar. By HH25, when species-specific morphologies are evident, p27 protein levels are dramatically different in the chicken and duck, in patterns consistent with alterations in miR-222 expression levels. These protein changes are not associated with changes in *p27* mRNA, indicating that post-transcriptional mechanisms (such as miRNA inhibition) are important for proper regulation of this cell cycle regulator.

While it is clear that changes in mRNA levels of the BMP/TGF-beta, calmodulin, and Wnt signaling pathways influence beak morphology [2], [3], [8], [9], [10], [20], and it is very likely that many more mRNAs differ across this developmental window, miRNAs add another layer to the regulation of species-specific morphogenesis. Our study provides the first insights into which specific miRNAs play roles in facial morphogenesis and the developmental processes that they may regulate.

## Materials and Methods

### miRNA isolation, sequencing, and analysis

Tissue and total RNA were isolated from the frontonasal mesenchyme of ducks, chickens and quails as previously described [3] for 40 or 5 embryos for the first and second biological samples, respectively. Fertilized duck (*Anas platyrhynchos domestica*), chicken (*Gallus gallus domesticus*), and quail (*Coturnix japonica*) eggs were obtained through AA Farms (Westminster, CA, USA) and incubated at 37°C until embryos reached stage 20 or stage 25 according to Hamburger-Hamilton criteria [19]. The FNP was dissected at both developmental stages by collecting the tissue rostral to the eyes and between the nasal pits. Mesenchyme—which, in the FNP, is a pure population of neural crest cells [29]—was isolated by incubating FNPs in 1.26 U dispase, and removing surface ectoderm and forebrain neuroectoderm using sharpened tungsten needles. Samples designated as “first biological sample” were derived from exactly the same total RNA samples previously analyzed for transcription factor gene expression [3]. Adapters were ligated to mature miRNAs using the Illumina Small RNA Sample Prep Kit per manufacturer's instructions (v1.5 sRNA 3′ Adapter). RNA species from 20–40 bp were size selected using a 6% Novex TBE Page gel (Invitrogen) and sequenced on a GAIIX platform (Illumina). Reads were mapped to known chicken and human mature miRNAs, allowing zero to two mismatches, using the miRanalyzer program (http://web.bioinformatics.cicbiogune.es/microRNA/miRanalyser.php, release version 1) [88]. For one sample, the second biological sample of HH25 chicken neural crest (Chick HH25 BS2), data from two replicate sequencing runs were combined after verifying that runs correlated >95% (data not shown). All sample preparation parameters and sequencing data are available through http://www.ncbi.nlm.nih.gov/geo/ under accession number GSE30716.

### Differential Expression

miRNAs were considered to be differentially expressed if they passed a >2-fold change and had a normalized read count of >15 PMMR in at least one library. IDEG6 software was used to determine statistically differentially expressed miRNAs within this set (http://telethon.bio.unipd.it/bioinfo/IDEG6/readme.html) [89]. Fisher's exact test (significance threshold <0.05) with a Bonferroni correction to account for multiple testing was implemented to calculate the p-values between libraries [90], [91]. DESeq [92] confirmed the fold change and significance trends (Table S6).

### Avian-specific miRNAs

A list was compiled of those mature miRNAs only annotated in miRBase (http://mirbase.org/, release version 16) [30] for chicken (*Gallus gallus*, WASHUC2 genome build) and/or zebrafinch (*Taeniopygia guttata*, taeGut3.2.4 genome build). Potential specificity to the avian lineage was assessed by BLAT analysis against genomic sequences of zebrafish (*Danio rerio*, danRer 7 genome build), lizard (*Anolis carolinensis*, anoCar1 genome build), frog (*Xenopus tropicalis*, xenTro2 genome build), *Caenorhabditis elegans* (ce6 genome build), *Drosophila melanogaster* (dm3 genome build), platypus (*Ornithorhynchus anatinus*, ornAna1 genome build), cow (*Bos Taurus*, bosTau4 genome build), dog (*Canis lupus familiaris*, canFam2 genome build), mouse (*Mus musculus*, mm9 genome build), and human (*Homo sapiens*, hg19 genome build). PCR was conducted on DNA from birds that span the avian lineage (Figure 2) [93]. Primers were designed against the mature and mature star strands of the pri-miRNA hairpin, avoiding the 5′ and 3′ nucleotides of each strand to account for their decreased conservation [76]. Species analyzed were Black-footed Albatross (*Phoebastria nigripes*), Common Nighthawk (*Chordeiles minor*), Green Warbler Finch (*Certhidea olivacea*), Groove-billed Ani (*Crotophaga sulcirostris*), Ostrich (*Struthio camelus*), Rhinoceros Hornbill (*Buceros bicornis*), and Tinamou (Spotted Nothura, *Nothura maculosa*).

### Quantitative real-time Polymerase Chain Reaction

Reverse transcription was performed with Taqman MicroRNA Reverse Transcription reagents (Applied Biosystems), and a quantitative real-time polymerase chain (qRT-PCR) reaction was carried out using the Applied Biosystems Prism 7500 per manufacturer's instructions. The levels of miRNA gene expression were determined by normalizing to the spliceosomal RNA *RNU6B*. All reactions were performed in triplicate.

### In situ hybridization

Chicken (*Gallus domesticus*, Charles River Labs) and duck (*Anas platyrhynchos*, Metzer Farms, Gonzales, CA) embryo heads were dissected in cold PBS and fixed in 4% paraformaldehyde in PBS overnight at 4°C. Embryos were serially dehydrated to 100% methanol for storage, and rehydrated in PBS before *in situ* hybridization. Whole mount *in situ* hybridization were then performed as previously described [3] on stage-matched embryos with 40 nM 5′ DIG-labeled miRCURY LNA probe (Exiqon).

### p27 Western

For western blotting, embryos were staged according to Hamburger-Hamilton criteria [19]. FNPs were isolated in cold PBS and lysed in 1× RIPA buffer supplemented with Complete Mini Protease Inhibitor Cocktail (Roche). Samples were resolved by 10% SDS/PAGE, transferred to nitrocellulose membrane, probed with mouse anti-p27^KIP1^ monoclonal antibody (BD Transduction Laboratories) and horseradish peroxidase-conjugated goat anti-mouse IgG (Sigma-Aldrich), and visualized by ECL (Pierce). The mouse anti-alpha-tubulin monoclonal antibody (Santa Cruz Biotechnology) was used as a loading control.

## Supporting Information

Figure S1 **Classification of Next-Generation short RNA sequencing (miRNA-seq) reads from all samples.** Reads are annotated as “mapped” if they can be located within the current version of the chicken genome (*Gallus gallus*, gga3 genome build).
(TIF)

Table S1 **miRNAs detectably expressed in avian frontonasal neural crest cells at HH20 and HH25.** MiRNAs expressed in chicken, quail, and ducks samples at a normalized read count of >15 PMMR in at least one sample. Genomic locations are mapped to the gga3 build of the *Gallus gallus* genome.
(XLS)

Table S2 **miRNAs differentially expressed among chicken, quail, and duck frontonasal neural crest cells.** Fold changes are on a log2 scale, with expression in duck relative to chicken or quail, in quail relative to chicken, or HH25 relative to HH20. For example, a negative number is expressed at a lower level in the duck versus chicken. Comparisons in bold typeface pass >2-fold change and normalized read count of >15 PMMR criteria. DC, duck/chicken comparison; DQ, duck/quail comparison; QC, quail/chicken comparison.
(XLS)

Table S3 **Complete list of predicted targets of miRNAs that are limited to the avian lineage.** Targets were predicted using TargetScan (http://www.targetscan.org/) and the seed sequence (nt 2–8) for each of the avian-specific miRNAs.
(XLS)

Table S4 **Complete list of enriched GO annotations of avian specific miRNAs.** Predicted targets of individual miRNA were further searched for GO annotation enrichment by the ToppGene software suite (http://toppgene.cchmc.org/). Due to the limited number of predicted downstream targets for gga-miR-1559 (two predicted targets, see Table S3) it was not included for enrichment analyses. Statistically significant GO annotations (p value<0.05 after Bonferroni correction) are listed for individual miRNAs.
(XLSX)

Table S5 **qRT-PCR validation of miRNA sequencing data.** Delta Ct (cycle threshold) values for all miRNAs relative to *RNU6B* input control. Note that values are on a log2 scale, with more positive values being more highly expressed.
(XLS)

Table S6 **DESeq analysis of differentially expressed miRNAs.** Accompanying each miRNA are fold changes (FC) on a log2 scale and a p-value. The analysis was conducted with DESeq [92] using default parameters with the following options used to estimate the dispersions: (1) the “fit-only” sharingMode was used for all datasets, (2) the “blind” method was used only for the quail dataset, and (3) a “local” fitType was used only when estimating dispersions with stage 25.
(XLSX)

Table S7 **Differentially expressed miRNAs with discernable trends among chicken, duck, and quail.** Accompanying each miRNA are fold changes (FC) on a log2 scale and Fisher's exact p-values (see Methods). The Bonferroni corrected threshold for significance is <1.97e-05. Values in the table that fail to reach this threshold are marked with ‡. For a complete list of differentially expressed miRNAs, see Table S2.
(XLSX)
