# Supplementary figures and images for: A Cross-Species Analysis of MicroRNAs in the Developing Avian Face
Source: PLoS One. 2012 Apr 16;7(4):e35111. doi: 10.1371/journal.pone.0035111 (PMC3327661; doi:10.1371/journal.pone.0035111)

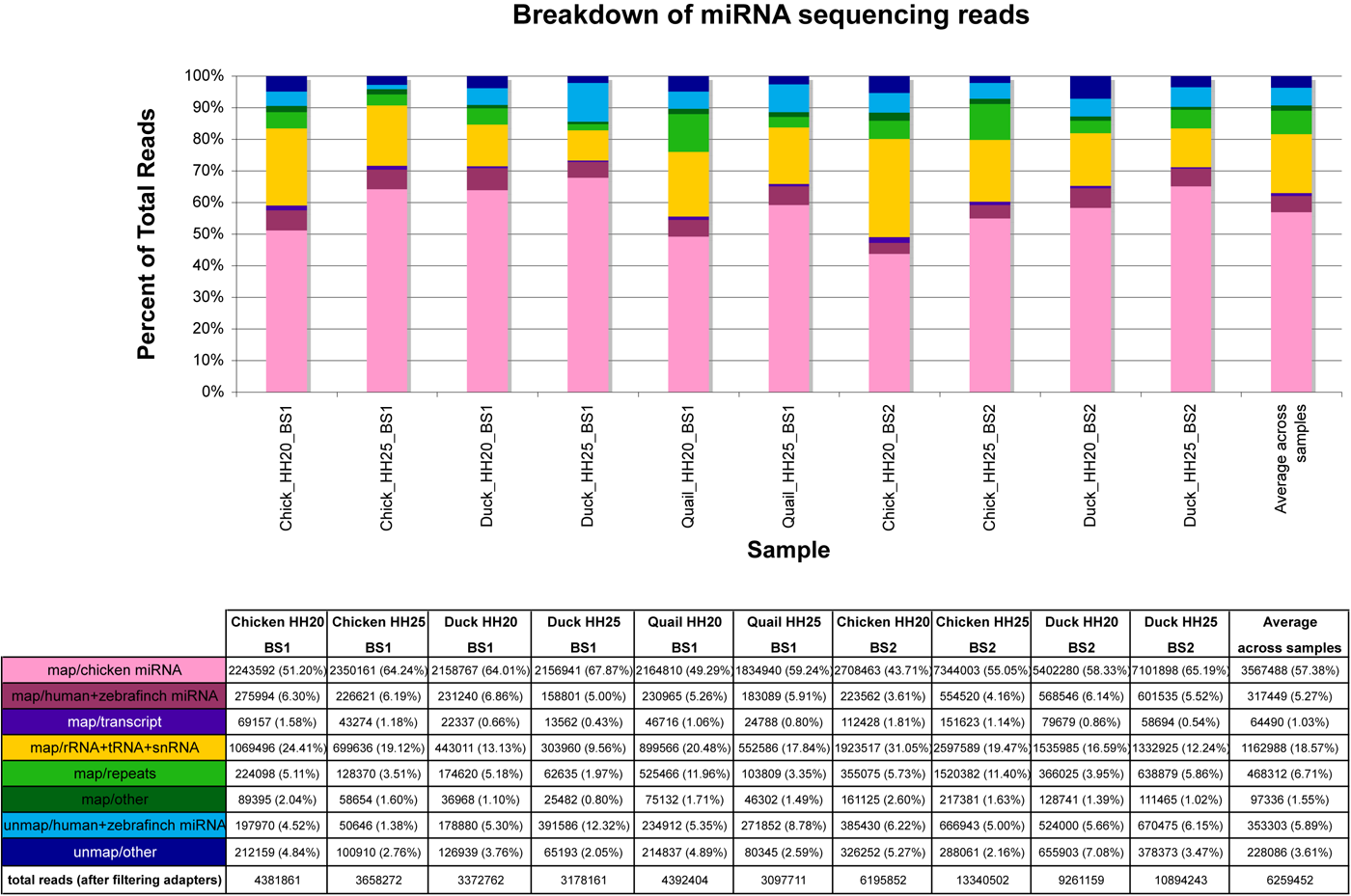

Supplement: Figure S1 — Classification of Next-Generation short RNA sequencing (miRNA-seq) reads from all samples. Reads are annotated as “mapped” if they can be located within the current version of the chicken genome (Gallus gallus, gga3 genome build). (TIF) [file pone.0035111.s001.tif]
